# Assessing organizational commitment among healthcare employees in the Asir Health Cluster, Saudi Arabia: a cross-sectional study

**DOI:** 10.3389/fmed.2026.1868245

**Published:** 2026-07-20

**Authors:** Mashael Taalae Alqahtani, Salah Alshagrawi

**Affiliations:** 1 Department of Command Center, Aseer Health Cluster, Aseer, Saudi Arabia; 2 Department of Public Health, College of Health Sciences, Saudi Electronic University, Riyadh, Saudi Arabia

**Keywords:** employee engagement, health systems transformation, healthcare workforce, organizational commitment, Saudi Arabia

## Abstract

**Background:**

Organizational commitment is a critical determinant of workforce stability, employee retention, and organizational performance in healthcare settings. As healthcare systems undergo large-scale transformation, maintaining a committed workforce becomes increasingly important for supporting service quality, organizational resilience, and successful implementation of reforms. In Saudi Arabia, the Health Sector Transformation Program has introduced integrated health cluster models that bring together hospitals, primary healthcare centers, and administrative services under unified governance. Despite the importance of organizational commitment in these evolving healthcare systems, evidence remains limited regarding commitment levels among employees working within integrated health clusters. Therefore, this study aimed to assess organizational commitment and identify associated factors among clinical and administrative employees in the Asir Health Cluster, Saudi Arabia.

**Methods:**

A cross-sectional study was conducted between October and December 2025 using the Meyer and Allen Organizational Commitment Scale. A total of 392 clinical and administrative employees participated. Descriptive statistics, independent-samples *t*-tests, and one-way analysis of variance (ANOVA) were used to examine differences in commitment levels. Binary logistic regression analysis was performed to identify independent predictors of organizational commitment.

**Results:**

Overall organizational commitment was moderate (*M* = 3.85, SD = 0.88). Normative commitment demonstrated the highest mean score (*M* = 3.98, SD = 1.07), followed by affective commitment (*M* = 3.84, SD = 0.87) and continuance commitment (*M* = 3.70, SD = 1.22). Commitment levels were generally similar across professional groups. Female employees were significantly more likely to report high organizational commitment than male employees (AOR = 2.45, 95% CI: 1.55–3.87, *p* < 0.001), while age and professional category were not significant predictors.

**Conclusion:**

Employees within the Asir Health Cluster demonstrated moderate organizational commitment, with commitment primarily driven by a sense of professional obligation and responsibility. The findings highlight opportunities to strengthen workforce engagement through initiatives that enhance affective commitment, including supportive leadership, employee recognition, and positive organizational culture. Organizational commitment should be considered a strategic workforce indicator within healthcare transformation efforts, and regular monitoring may support workforce planning, employee retention, and the long-term sustainability of health system reforms under Saudi Vision 2030.

## 1 Introduction

The healthcare workforce is the cornerstone of effective health system performance, as the quality, continuity, and safety of healthcare services depend heavily on employees’ commitment and engagement (1). Globally, healthcare organizations operate in increasingly complex environments characterized by rising service demands, workforce shortages, and continuous organizational reforms (2). In such contexts, understanding employee attitudes toward their organizations has become a critical priority for healthcare leaders and policymakers, particularly organizational commitment due to its strong association with workforce stability, performance, and organizational effectiveness (3, 4).

Organizational commitment refers to the psychological bond that connects employees to their organization and influences their willingness to remain and contribute to organizational goals (5). High levels of commitment are associated with improved job performance, reduced turnover intentions, and enhanced organizational cohesion, whereas low commitment may result in disengagement, absenteeism, and increased staff turnover, ultimately affecting healthcare delivery and patient outcomes (6, 7). Given the labor-intensive nature of healthcare services, maintaining a committed workforce is essential for sustaining service quality and organizational resilience (8).

The concept of organizational commitment has evolved within organizational behavior research from a unidimensional construct reflecting employees’ identification with organizational goals to a multidimensional psychological state shaped by individual, cultural, and structural factors (1–3, 6). One of the most widely adopted frameworks is the Three-Component Model proposed by Meyer and Allen, which conceptualizes commitment as comprising affective, continuance, and normative dimensions (9). Affective commitment reflects emotional attachment, continuance commitment relates to perceived costs of leaving, and normative commitment represents a sense of obligation to remain. This model provides a comprehensive framework for understanding employee–organization relationships and has been extensively applied in healthcare settings, where emotional, ethical, and practical considerations often coexist (1).

In healthcare environments, organizational commitment is particularly important due to the demanding nature of work and its direct impact on service delivery (3, 10). Healthcare professionals frequently operate under high workloads, emotional stress, and ethical responsibilities to patients and communities (11). Strong commitment has been associated with improved teamwork, continuity of care, and organizational stability. Importantly, organizational commitment extends beyond clinical roles, as administrative employees also play a critical role in planning, coordination, and policy implementation within healthcare systems (2, 3).

In Saudi Arabia, the healthcare sector is undergoing significant transformation under Saudi Vision 2030, a national strategy aimed at enhancing the quality of life, improving health outcomes, and ensuring the sustainability and efficiency of healthcare services (12). Central to this transformation is the Health Sector Transformation Program, which seeks to establish an integrated, patient-centered healthcare system through improved service quality, resource optimization, and digital innovation (13, 14). The success of these reforms depends not only on structural and technological changes but also on the healthcare workforce, which serves as the operational foundation for implementing policies and delivering care (15).

Within this context, organizational commitment plays a crucial role in influencing employee engagement, performance, and adaptability during periods of change. High levels of commitment are associated with improved job performance, reduced resistance to change, increased job satisfaction, and enhanced organizational stability (16). Employees with strong commitment are more likely to support transformation initiatives, adopt new practices, and contribute to achieving organizational and national healthcare objectives, whereas low commitment may hinder reform efforts and negatively affect service quality (17, 18).

Despite its importance, empirical research on organizational commitment within integrated healthcare systems remains limited. Most existing studies have focused on specific professional groups or individual institutions, with less attention given to broader organizational structures such as health clusters that integrate multiple facilities and professional categories under unified governance (19, 20). This gap highlights the need to examine organizational commitment across both clinical and administrative employees within such systems, particularly in the context of ongoing healthcare transformation in Saudi Arabia.

Addressing this gap is essential for understanding workforce dynamics and supporting evidence-based decision-making. Assessing organizational commitment at the health cluster level can provide baseline insights to inform workforce planning, organizational development, and policy formulation, particularly in regional contexts where organizational structures and workforce characteristics may differ (21).

The novelty of this study lies in its focus on organizational commitment within a health cluster model, an emerging form of healthcare governance that integrates hospitals, primary healthcare centers, and administrative services under a unified management structure. While previous studies have largely examined organizational commitment within single institutions or among specific professional groups, limited evidence is available regarding workforce commitment across integrated healthcare systems. Furthermore, most existing studies have focused primarily on clinical personnel, with relatively little attention given to administrative employees despite their important role in healthcare operations and organizational performance. By examining both clinical and administrative employees within a large regional health cluster undergoing healthcare transformation, this study extends the current literature and provides a more comprehensive understanding of organizational commitment within integrated healthcare environments. The findings contribute new evidence relevant to workforce planning, organizational development, and the successful implementation of health system reforms under Saudi Vision 2030.

Therefore, this study aims to assess the level of organizational commitment among clinical and administrative employees in the Asir Health Cluster. By adopting a quantitative cross-sectional design and utilizing a validated measurement framework, the study provides a comprehensive evaluation of organizational commitment and its dimensions within an integrated healthcare system. The findings are expected to contribute both theoretically, by addressing gaps in the literature on organizational commitment in Saudi healthcare, and practically, by informing strategies to enhance workforce stability, employee engagement, and the sustainability of healthcare transformation initiatives.

## 2 Methods

This section describes the methodology used in the present study. It outlines the study design, setting, participants, sampling strategy, data collection tools, and statistical analysis procedures.

### 2.1 Study design

This study employed a quantitative cross-sectional descriptive research design. A cross-sectional design was chosen because it allows assessment of organizational commitment among employees. This design is appropriate for describing the current level and dimensions of organizational commitment within the Asir Health Cluster.

### 2.2 Setting

The study was conducted in the Asir Health Cluster, Saudi Arabia. The cluster includes 29 hospitals, 271 primary healthcare centers, and administrative units that support healthcare service delivery across the region. The Asir Health Cluster operates as an integrated healthcare system under unified governance and serves a large and diverse population. Data collection was carried out between October and December 2025. During this period, eligible employees across clinical and administrative units were invited to participate in the study.

### 2.3 Participants

Eligibility Criteria and Selection of Participants: The study population included clinical and administrative employees working in the Asir Health Cluster. Clinical staff comprised physicians, nurses, and other healthcare professionals, while administrative staff included employees involved in managerial, operational, and support roles. Participants were eligible if they were aged 18 years or older and currently employed within the cluster at the time of data collection. Participants were recruited through convenience sampling. The questionnaire was distributed electronically through internal communication channels, and participation was voluntary.

The total workforce of the Asir Health Cluster is estimated to be approximately 22,000 employees. The required sample size was calculated using Cochran’s sample size formula for proportions, assuming a population proportion of 50%, a 95% confidence level, and a margin of error of 5%. Based on this calculation, the minimum required sample size was estimated to be approximately 385 participants. A total of 392 complete responses were obtained and included in the final analysis, exceeding the minimum required sample size.

### 2.4 Data measurement

The primary outcome variable in this study was organizational commitment, measured as a multidimensional construct consisting of affective, continuance, and normative commitment. Different variables, such as age group, gender, and professional category, were treated as independent variables for subgroup comparisons to explore differences in organizational commitment levels across groups.

Data were obtained directly from participants through a self-administered electronic questionnaire. Organizational commitment was measured using standardized items across the three commitment domains. All participants completed the same questionnaire, ensuring comparability of assessment methods across clinical and administrative groups.

The measurement tool used in this study was a structured questionnaire consisting of two sections. The first section collected demographic information, while the second section assessed organizational commitment using the Organizational Commitment Scale. The questionnaire was developed based on the Organizational Commitment Scale proposed by Meyer and Allen (22). The scale includes items measuring affective, continuance, and normative commitment. All items were presented in a Likert-type format to capture participants’ level of agreement. The Organizational Commitment Scale used in this study is a widely validated instrument that has been employed extensively in prior research across diverse organizational and cultural contexts. The internal consistency of the Organizational Commitment Scale was assessed using Cronbach’s alpha. The overall scale demonstrated good reliability (*α* = 0.88). The reliability coefficients for the subscales were as follows: affective commitment (*α* = 0.86), continuance commitment (*α* = 0.78), and normative commitment (*α* = 0.84).

Potential selection bias may have occurred due to the voluntary nature of participation and the use of convenience sampling. To minimize this risk, the questionnaire was distributed widely across different departments and professional groups within the cluster. Reminders were used to encourage participation and enhance representation from both clinical and administrative employees.

### 2.5 Statistical analysis

Data were analyzed using IBM SPSS Statistics for Windows, Version 29.0 (IBM Corp., Armonk, NY, United States). Descriptive statistics were used to summarize participant characteristics and organizational commitment scores. Categorical variables were presented as frequencies and percentages, while continuous variables were summarized using means and standard deviations. Group comparisons were conducted, where applicable, using independent-samples *t*-tests for two-group comparisons and one-way analysis of variance (ANOVA) for comparisons involving more than two groups.

In addition to descriptive and bivariate analyses, binary logistic regression analysis was conducted to identify independent predictors of organizational commitment. Because no universally accepted cutoff values exist for categorizing organizational commitment using the Meyer and Allen Organizational Commitment Scale, the overall organizational commitment score was dichotomized into “high commitment” and “low commitment” using the sample median as the cutoff point. The median split approach was selected to create two approximately equal comparison groups and facilitate logistic regression analysis. Similar approaches have been used in organizational and behavioral research when established clinical or theoretical thresholds are unavailable. Variables with *p* < 0.20 in bivariate analysis were entered into the multivariable logistic regression model. Adjusted odds ratios (AORs) with 95% confidence intervals (CIs) were reported. All statistical tests were two-tailed, with a significance threshold set at the 5% alpha error level (*p* < 0.05).

## 3 Results

### 3.1 Participants

**Figure 1:**
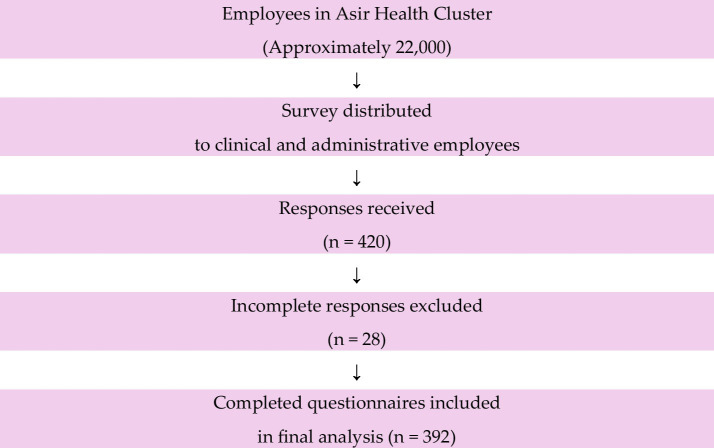
Flowchart of the sampling process.

Approximately 22,000 employees working in the Asir Health Cluster were considered potentially eligible to participate in the study. The questionnaire was distributed to clinical and administrative employees through internal communication channels. A total of 420 responses were received. Of these, 28 responses were excluded due to incomplete data. As a result, 392 complete questionnaires were included in the final analysis. The sampling and participant selection process is illustrated in Figure 1.

### 3.2 Demographic characteristics of participants

Table 1 shows the demographic characteristics of participants. A total of 392 participants were included in the analysis from both clinical and administrative divisions. The administrative staff constitutes the largest group, 142 (36.2%), followed by nurses, 109 (27.8%), other healthcare staff, 92 (23.5%), and physicians, 94 (12.5%). Most participants were aged 25–34 years (213, 54.3%), followed by those aged 35–44 years (144, 36.7%). Most participants were male (n = 257, 65.6%), and they had an average of 8.7 years (SD = 5.2) of professional experience.

**Table 1:** Demographic characteristics of participants.

Variable	*n* (%)
Gender	
Male	257 (65.6%)
Female	135 (34.4%)
Profession	
Physician	49 (12.5%)
Nurse	109 (27.8%)
Administrative	142 (36.2%)
Other	92 (23.5%)
Age group (years)	
18–24	0 (0.0%)
25–34	214 (54.6%)
35–44	144 (36.7%)
45–54	29 (7.4%)
≥55	5 (1.3%)

### 3.3 Levels of organizational commitment and its subscales

The outcome of interest in this study was organizational commitment. Overall organizational commitment and its three dimensions, affective, continuance, and normative commitment, were summarized using mean scores and standard deviations. The results reflect the current level of organizational commitment among clinical and administrative employees.

Table 2 summarizes the mean and standard deviation for the organizational commitment domains. Overall organizational commitment was moderate (*M* = 3.85, SD = 0.88). Among the three domains, normative commitment had the highest mean score (*M* = 3.98, SD = 1.07), followed by affective commitment (*M* = 3.84, SD = 0.87), while continuance commitment recorded the lowest mean score (*M* = 3.70, SD = 1.22).

**Table 2:** Mean and standard deviation of organizational commitment domains.

Commitment Domain	*N*	Mean	SD
Affective commitment	392	3.84	0.87
Continuance commitment	392	3.70	1.22
Normative commitment	392	3.98	1.07
Overall organizational commitment	392	3.85	0.88

Table 3 shows the reported organizational commitment among clinical and administrative employees across professional groups. The commitment levels were relatively similar among physicians (*M* = 3.85) (SD = 0.95), nurses (*M* = 3.86) (SD = 0.91), administrative staff (*M* = 3.85) (SD = 0.84), and other healthcare employees (*M* = 3.82) (SD = 0.87). Across all groups, normative commitment had the highest mean score, whereas continuance commitment had the lowest, indicating consistent patterns of commitment across occupational categories.

**Table 3:** Descriptive statistics of organizational commitment domains across professional groups.

Group	*N*	Affective mean ± SD	Continuance mean ± SD	Normative mean ± SD	Overall mean ± SD
Physician	94	3.85 ± 1.05	3.72 ± 1.38	3.98 ± 1.21	3.85 ± 0.95
Nurse	109	3.84 ± 0.89	3.73 ± 1.25	3.98 ± 1.13	3.86 ± 0.91
Other healthcare	92	3.83 ± 0.87	3.69 ± 1.28	3.94 ± 1.09	3.82 ± 0.87
Administrative	142	3.84 ± 0.78	3.73 ± 1.08	3.98 ± 0.97	3.85 ± 0.84

### 3.4 Factors associated with organizational commitment

Table 4 presents the association between demographic variables and organizational commitment level (committed vs. not committed). A statistically significant association was observed between gender and organizational commitment, with female employees demonstrating higher organizational commitment than male employees (*p* < 0.001). No statistically significant associations were found between organizational commitment and age group (*p* = 0.712) or professional category (*p* = 0.530).

**Table 4:** Factors associated with organizational commitment.

Variable	Category	Committed *n* (%)	Not Committed *n* (%)	*p*-value
Age Group	18–34	67.8%)(145	32.2%)(69	0.712
	35–44	70.1%)(101	29.9%)(43	
	45–≤55	69.7%)(23	30.3%)(10	
Gender	Female	76.3%)(196	23.7%)(61	<0.001
	Male	54.8%)(74	45.2%)(61	
Professional category	Physician	61.2%)(30	38.8%)(19	0.530
	Nurse	67.0%)(73	33.0%)(36	
	Other Healthcare	71.7%)(66	28.3%)(26	
	Administrative	71.1%)(101	28.9%)(41	

Binary logistic regression analysis was performed to identify independent predictors of organizational commitment (Table 5). After adjusting for age group, gender, and professional category, gender remained a statistically significant predictor of organizational commitment. Female employees were more likely to report high organizational commitment compared to male employees (AOR = 2.45, 95% CI: 1.55–3.87, *p* < 0.001).

**Table 5:** Multivariable logistic regression analysis of factors associated with organizational commitment.

Variable	Category	AOR	95% CI	*p*-value
Gender	Male (Ref)	1.00	–	–
	Female	**2.45**	1.55–3.87	**<0.001**
Age group	18–34 (Ref)	1.00	—	–
	35–44	1.12	0.70–1.78	0.62
	45–≥55	1.05	0.48–2.28	0.89
Profession	Physician (Ref)	1.00	–	–
	Nurse	1.28	0.68–2.39	0.44
	Other Healthcare	1.46	0.75–2.83	0.26
	Administrative	1.39	0.74–2.61	0.30

Bold values indicate statistically significant associations at the 5% significance level (*p* < 0.05).

No statistically significant associations were observed for age group or professional category in the adjusted model (*p* > 0.05). These findings indicate that gender is an independent factor associated with organizational commitment in this study population.

## 4 Discussion

This study assessed organizational commitment among clinical and administrative employees in the Asir Health Cluster and examined its distribution across the affective, continuance, and normative dimensions. The findings provide important information on the current state of employee commitment within a regional health cluster where workforce stability is a critical determinant of healthcare.

The findings indicate a moderate level of organizational commitment among employees in the Asir Health Cluster, suggesting a reasonable degree of organizational attachment while highlighting opportunities for further workforce engagement. The predominance of normative commitment indicates that employees’ connection to the organization is strongly influenced by professional responsibility and ethical obligations, a pattern commonly reported in healthcare settings where commitment extends beyond purely economic considerations (1, 21). While affective commitment was also evident, its comparatively lower level suggests opportunities for healthcare leaders to strengthen employees’ emotional attachment through supportive leadership, recognition, and positive organizational culture (19). These findings are particularly relevant within the context of healthcare transformation, where workforce commitment plays a critical role in supporting organizational change and service improvement.

The findings of this study are generally consistent with previous research in healthcare settings, which frequently reports moderate levels of organizational commitment among healthcare workers (19, 20). International studies have also shown that normative commitment often plays a prominent role in healthcare environments, driven by strong professional values and ethical obligations (21). However, most existing studies have focused on individual hospitals or specific professional groups such as nurses or physicians. In contrast, this study examined organizational commitment within a health cluster structure that integrates multiple hospitals and primary healthcare centers under a unified governance framework. This organizational model is relatively new in Saudi Arabia and is part of the broader Health Sector Transformation Program (13).

Additionally, limited research has examined organizational commitment among both clinical and administrative employees within the same healthcare system. By including both groups, this study contributes to a broader understanding of workforce commitment across diverse professional roles, supporting calls for more system-level investigations in integrated healthcare models (23).

The findings are also consistent with recent international studies conducted in healthcare settings outside Saudi Arabia. Research from Ethiopia, Turkey, and primary healthcare systems in Europe has similarly reported moderate levels of organizational commitment among healthcare professionals, with affective and normative commitment frequently emerging as prominent dimensions of commitment (19–21). Previous studies have suggested that organizational commitment is influenced by factors such as organizational support, workplace culture, leadership practices, and opportunities for professional development (5, 16, 17). Although the overall level of commitment observed in the present study is comparable to those reported internationally, the health cluster context introduces unique organizational characteristics related to integrated governance, workforce restructuring, and healthcare transformation. Consequently, the present findings contribute additional evidence regarding employee commitment within large-scale healthcare reform environments and extend the limited literature available on organizational commitment in integrated healthcare systems (13, 19).

The findings of this study have several practical implications for healthcare leaders and policymakers within the Asir Health Cluster and similar healthcare systems. Understanding the current level and pattern of organizational commitment can help decision-makers identify areas where workforce engagement strategies can be strengthened. For example, the relatively higher normative commitment suggests that employees feel a strong sense of obligation, reflecting strong professional ethics; however, efforts to enhance affective commitment may further improve job satisfaction, motivation, and long-term retention (19). From a management perspective, fostering a supportive organizational culture, promoting transparent communication, and recognizing employee contributions may help strengthen emotional attachment to the organization. Evidence suggests that positive leadership practices and supportive work environments are associated with stronger organizational commitment in healthcare settings (21). At the policy level, these findings provide baseline data that can inform workforce planning initiatives aligned with Saudi Vision 2030 and the Health Sector Transformation Program (24). Monitoring organizational commitment over time may help assess the impact of structural reforms on employee attitudes and organizational stability. Importantly, organizational commitment may serve as a strategic indicator of workforce stability during periods of healthcare transformation. Continuous monitoring of this indicator can provide early signals of organizational resilience or vulnerability, enabling proactive leadership interventions before workforce instability manifests in turnover, disengagement, or reduced performance. Integrating commitment metrics into workforce dashboards and institutional performance frameworks could therefore enhance reform governance and early risk detection mechanisms. In terms of future research, longitudinal studies could examine changes in organizational commitment as the health cluster model matures. Future studies may also explore determinants of commitment, such as leadership styles, organizational culture, and job satisfaction, to better understand the mechanisms underlying employee attachment (25).

An important finding of this study was the significant association between gender and organizational commitment, with female employees demonstrating higher odds of reporting high organizational commitment than male employees. Similar findings have been reported in some healthcare settings, although the evidence remains mixed across countries and professional groups (19, 21). One possible explanation is that female healthcare employees may develop stronger affective and normative connections to their organizations through professional values, interpersonal relationships, and organizational engagement. Previous research has suggested that organizational support, workplace relationships, and job satisfaction are important determinants of organizational commitment and may influence employee attachment differently across demographic groups (5, 16, 21). Additionally, women may place greater emphasis on collaborative work environments and social aspects of organizational life, which could contribute to stronger organizational attachment in healthcare settings (21). Nevertheless, gender differences in organizational commitment are influenced by complex cultural, occupational, and organizational factors and should therefore be interpreted cautiously. Because the present study was not designed to investigate the mechanisms underlying this association, future research is needed to explore the factors contributing to gender-related differences in organizational commitment within integrated healthcare systems (26).

## 5 Conclusion

This study examined organizational commitment among clinical and administrative employees in the Asir Health Cluster, highlighting it as a key workforce indicator for healthcare system transformation in Saudi Arabia. Organizational commitment remains a critical element for ensuring workforce stability, maintaining service quality, and supporting the effective implementation of organizational reforms. The findings indicate that employees in the Asir Health Cluster demonstrate a moderate level of overall organizational commitment, with normative commitment emerging as the most prominent dimension. This suggests that employees’ attachment to the organization is largely driven by a sense of duty and moral obligation rather than by purely emotional attachment or by the perceived costs of leaving. Such a pattern is particularly relevant in healthcare settings, where professional ethics and responsibility toward patient care strongly influence employee attitudes and behaviors. The results of this study suggest significant potential to strengthen employee engagement, particularly by enhancing affective commitment. Strengthening emotional attachment to the organization may improve motivation, job satisfaction, and long-term retention, which are essential outcomes in a sector facing increasing demands and workforce pressures. Finally, this study supports the need for healthcare leaders and policymakers to prioritize organizational commitment within broader workforce development strategies. By understanding employees’ current commitment profile, healthcare organizations can design targeted initiatives that promote a supportive work environment and reinforce employees’ connection to their organization.

## 6 Limitations

This study has several limitations that should be considered when interpreting the findings. First, the use of convenience sampling and voluntary participation may have introduced selection bias and limited the representativeness of the sample. Employees who were more engaged with their organization, more interested in workplace issues, or more willing to participate in organizational surveys may have been more likely to respond, potentially resulting in an overestimation of organizational commitment levels. Conversely, employees with lower commitment, greater dissatisfaction, or heavier workloads may have been less likely to participate. Although the survey was distributed widely across clinical and administrative departments and reminders were used to encourage participation, the final sample may not fully reflect the characteristics and perceptions of all employees within the Asir Health Cluster. Therefore, caution should be exercised when generalizing the findings to the entire workforce or to other healthcare settings.

Second, the cross-sectional design limits the ability to establish causal relationships between variables. The study captures organizational commitment at a single point in time; therefore, changes in commitment levels over time or in response to organizational reforms cannot be assessed.

Third, data were collected using a self-administered online questionnaire, which may be subject to response bias and social desirability bias. Participants may have provided responses that reflected perceived expectations rather than their actual experiences or opinions.

Finally, the study focused primarily on organizational commitment and did not examine other potentially important determinants, such as leadership style, organizational culture, work environment, or job satisfaction. Future studies incorporating these factors may provide a more comprehensive understanding of organizational commitment within integrated healthcare systems.

## 7 Recommendations

Based on the findings of this study, several practical recommendations can be proposed to support workforce stability and strengthen organizational commitment within the Asir Health Cluster. Given that overall organizational commitment was moderate, with normative commitment as the most prominent dimension, targeted efforts are needed to strengthen employees’ emotional attachment and long-term engagement with the organization.

First, healthcare leaders and administrators are encouraged to focus on initiatives that strengthen employees’ affective commitment. This may include fostering a supportive work environment, promoting transparent communication, and recognizing employee contributions. Providing regular feedback and acknowledging professional efforts can help employees feel valued and emotionally connected to the organization, thereby enhancing motivation and job satisfaction.

Second, organizational policies should prioritize employee engagement strategies that align with the health cluster’s values and mission. Clear articulation of organizational goals and reinforcing a shared sense of purpose may further strengthen employees’ sense of responsibility and belonging. Given the relatively high normative commitment, reinforcing ethical values and professional responsibility through training and organizational culture initiatives may help sustain this positive dimension of commitment.

Third, policymakers and decision-makers within the healthcare system should consider integrating organizational commitment indicators into workforce planning and performance evaluation frameworks. Monitoring commitment levels over time can provide valuable insights into the impact of organizational changes and reforms, particularly within the context of health cluster implementation and the Health Sector Transformation Program.

Fourth, investing in leadership development programs is recommended to ensure managers and supervisors are equipped with the skills to support and engage their teams effectively. Although this study did not examine specific predictors of commitment, existing evidence suggests that leadership practices play a key role in shaping employee attitudes and organizational attachment.

Finally, future research is recommended to build on this study’s findings by exploring factors influencing organizational commitment, such as leadership styles, organizational culture, and the work environment. Longitudinal studies may also be conducted to examine changes in commitment over time and to assess the long-term impact of organizational reforms within health clusters.

## Data Availability

The raw data supporting the conclusions of this article will be made available by the authors, without undue reservation.
